# Impact of ambient temperature on adverse pregnancy outcomes: a birth cohort study in Fuzhou, China

**DOI:** 10.3389/fpubh.2023.1183129

**Published:** 2023-07-06

**Authors:** Jinfeng Lin, Yan Yang, Ayinasaer Nuermaimaiti, Tingting Ye, Jingwen Liu, Zitong Zhang, Yifeng Chen, Qingyu Li, Chuancheng Wu, Baoying Liu, Rongxian Xu, Yong Xia, Jianjun Xiang

**Affiliations:** ^1^Fujian Center for Prevention and Control of Occupational Diseases and Chemical Poisoning, Fuzhou, Fujian, China; ^2^Department of Preventive Medicine, School of Public Health, Fujian Medical University, Fuzhou, Fujian, China; ^3^Climate, Air Quality Research Unit, School of Public Health and Preventive Medicine, Monash University, Melbourne, VIC, Australia; ^4^School of Public Health, The University of Adelaide, Adelaide, SA, Australia; ^5^Department of Nutrition and Food Safety, School of Public Health, Fujian Medical University, Fuzhou, Fujian, China; ^6^Fuzhou Maternity and Child Health Care Hospital, Fuzhou, Fujian, China

**Keywords:** ambient temperature, adverse pregnancy outcome, pregnancy complication, neonatal jaundice, Fuzhou

## Abstract

**Background:**

Previous studies have identified a series of specific adverse pregnancy outcomes (APOs) linked with temperature extremes. Most of them focus on preterm birth, low birth weight, and stillbirth. Other possible adverse outcomes were under-researched. This study aimed to investigate the impact of ambient temperature on maternal complications, white blood cell count (WBC), newborn hearing, and neonatal jaundice.

**Methods:**

A total of 418 participants were recruited from Fuzhou Maternity & Child Healthcare Hospital in 2016. Participants were invited to fill out a structured questionnaire. The gridded near-surface air temperatures at a resolution of 0.1°* 0.1° for Fuzhou were extracted from a published dataset. Meteorological data and PM_2.5_ were extracted based on participants’ residential addresses using R packages “ncdf4” and “raster.” Multivariate logistic regression models were used to quantify the effects of ambient temperature on APOs after controlling for confounders.

**Results:**

Overall, there were 107 APOs, accounting for 25.6% of all participants. Every 1°C increase in mean temperature was associated with a 10.0% increase in APOs (aOR = 1.100, 95%CI 1.006–1.203) during the period of early pregnancy. However, negative associations were observed in the middle pregnancy period, and a 1°C increase in mean temperature was associated 8.8% decrease in APOs (aOR = 0.912, 95%CI 0.846–0.982). Diurnal temperature variation had a significant impact on APOs in the third trimester. Infant jaundice was negatively associated with temperature exposure in the middle and late pregnancy periods. The risk of neonatal jaundice increased at lag weeks 2–9 in the first trimester, with the greatest lagged effect (aOR = 1.201, 95%CI 1.020–1.413) observed at lag week 3. A 1°C increase in mean temperature led to a 29.6% (aOR = 1.296, 95%CI 1.019–1.649) increase in high WBC. A 1°C increase in temperature variation was associated with more than two times (aOR = 2.469, 95%CI 1.001–6.089) increase of high WBC in the first trimester and about five times (aOR = 4.724, 95%CI 1.548–14.409) increase in the third trimester.

**Conclusion:**

Ambient temperature affects neonatal jaundice, newborn hearing loss, and infections during pregnancy. In addition to the identified epidemiologic link and susceptible exposure windows, there is a need to understand the underlying biological mechanisms for better recommendations for climate change adaptation policies.

## 1. Introduction

Adverse pregnancy outcomes (APOs), mainly including gestational diabetes, hypertensive disorders of pregnancy, preterm birth, low birth weight, stillbirth, premature rupture of membrane, and birth defect, are important public health concerns worldwide (1), affecting up to 16% of pregnancies in China during 2007–2019 (2). In some low-income countries such as Ethiopia, the prevalence of APOs could reach as high as 28% (3). Climate change has been identified as the single biggest health threat facing humanity and is predicted to cause about 250,000 additional death per year globally in the coming decades (4). Evidence has shown that climate-sensitive health risks disproportionately affect the most vulnerable and disadvantaged sub-groups (e.g., older adults, those with chronic diseases, and outdoor labor workers), especially in some low- and middle-income countries (5).

Pregnant women are vulnerable to extreme temperatures as they experience pregnancy-related physiological and psychological changes associated with thermoregulation mechanisms (6), such as increased body weight in a short period of time, decreased ratio of surface area to body mass, and fetal metabolic heat. Epidemiological evidence has shown that the impact of climate change may not only be limited to the maternal period but also extend to an individual’s lifespan and even into future generations (1, 7). However, pregnant women and the growing fetus have not been regarded as being vulnerable to climate change-related extreme temperatures until recently (7–9). In recognition of the need to prepare for the likely increasing climate crisis, more and more researchers call for awareness and global actions to respond to the maternal and newborn health risks because of climate change (10, 11). Since 2020, the International Federation of Gynecology and Obstetrics has taken actions by incorporating climate change into its education, advocacy, and research programs (9).

APOs are generally considered induced by the combined effects of genetic, behavioral, socioeconomic, and environmental risk factors. Climate change is one of the most important environmental stressors in this century. Many previous studies have identified a series of specific APOs linked with temperature extremes (12–14), and most of them focus on preterm birth, low birth weight, stillbirth, and premature rupture of membrane. By contrast, other possible maternal, fetal, and neonatal adverse outcomes were neglected in the global literature (6, 15). In this study, we investigated the impact of ambient temperature on maternal complications, high white blood cell (WBC), newborn hearing loss, and neonatal jaundice. Findings of this study may provide further evidence for a better understanding of the mechanisms of temperature-APOs and inform adaptation measures for women’s healthcare providers, the public, and policymakers to reduce climate change-attributable APOs.

## 2. Materials and methods

### 2.1. Participant recruitment

The study participants were recruited from the antenatal care outpatient units of Fuzhou Maternity & Child Healthcare Hospital in the metro area and another two hospitals in the suburban counties (Fuqing Women & Children Hospital, Changle Hospital) of Fuzhou City, the Capital of Fujian Province between January and December in 2016. Characterized with long and hot summers, Fuzhou has a humid subtropical climate, with a population of around 8 million in 2020. The inclusion criteria were hCG or/and ultrasound-confirmed clinical pregnancy, maternal age between 18–50 years, without the following diseases which were found to be associated with APOs: hyperthyroidism (16), heart disease (17), chronic kidney disease (18), tuberculosis (19), and psychiatric disease (20). Those who did not live in Fuzhou city during pregnancy and gestational age ≥ 42 weeks were excluded from this study. Participants were invited to fill out a structured questionnaire through face-to-face interviews. Participation was completely voluntary. A written informed consent form was obtained from each participant at enrolment. Ethical approvals were obtained from the Ethics Committees of above mentioned three hospitals (approval numbers: 20151110FQ, 20151110FZ, and 20,160,307).

### 2.2. Outcome definition

In this study, APOs include adverse maternal outcomes (gestational diabetes, gestational hypertension, and premature rupture of membranes), birth defect, preterm birth, failed newborn hearing test, neonatal jaundice, low birth weight, and high WBC count. We divided the entire pregnancy into three stages: early pregnancy (0–13 weeks, 1st trimester), middle pregnancy (14–26 weeks, 2nd trimester), and the last stage from week 27 to the delivery (3rd trimester).

### 2.3. Exposure assessment

The gridded near-surface air temperatures at a resolution of 0.1°* 0.1° for Fuzhou in 2015–2016 were downloaded from a published dataset (21), including daily average, maximum, and minimum temperatures. This dataset was modeled using the China Meteorological Forcing Dataset (CMFD) and ERA5 reanalysis data. CMFD is a set of comprehensive meteorological datasets developed by the Institute of Tibetan Plateau Research, Chinese Academy of Sciences, with a temporal resolution of 3 h and a spatial resolution of 0.3° (22). ERA5 is the 5th-generation product of the atmospheric reanalysis of global climate data launched by the European Center for Medium-Range Weather Forecast, with a temporal resolution of 1 h and a spatial resolution of 0.1° (23).

Gridded daily air pollution data (e.g., PM_2.5_) at a spatial resolution of 1 kilometer were downloaded from a publicly available dataset (ChinaHighPM_2.5_).^1^ These data were generated from multiple data sources including ground-based measurements, satellite remote sensing products, atmospheric reanalysis, and model simulations, using artificial intelligence by considering the spatiotemporal heterogeneity of air pollution (24). Missing values of daily temperature and PM_2.5_ were interpolated using inverse distance weighted (IDW) models (by using “gstat” package in R4.2) (25).

In this study, a two-step method was used to estimate each individual prenatal exposure to ambient temperature and PM_2.5_. First, we used Baidu Map API^2^ to transform participants’ home addresses into longitude and latitude coordinates. The participants’ locations were reprojected to WGS 84 using “geoChina” package in R. Then, daily mean, maximum, and minimum temperatures, and PM_2.5_ were extracted from the gridded datasets using R packages “ncdf4” and “raster.” Diurnal temperature variation is the variation between the maximum and minimum temperatures during the same day. The average exposure values of trimester-specific temperatures and PM_2.5_ were calculated for each participant.

### 2.4. Statistical analysis

The demographic characteristics of participants were descriptively summarized as counts and percentages or as mean and standard deviation (SD) for categorical variables and continuous variables, respectively. Bivariate logistic regression analysis was used to compare the differences in adverse pregnancy outcomes by different covariates. T-test was conducted to compare maternal weight and baby weight between APOs and non-APOs. Stata command ‘pwmean’ was used to make pairwise comparisons in temperature exposure and air pollution between the three trimesters. In the multivariate logistic regression model, we adjusted the following confounding factors: maternal age, maternal education level, maternal infection, household income, mode of delivery, location (metro or suburb), infant sex, parity, and gravidity. As previous evidence has shown that air pollutants may affect pregnancy outcomes (26), PM_2.5_ was put into the regression model as a proxy of air pollution and controlled for as a potential confounder.

In addition to the overall temperature-APOs association at each trimester, we also conducted APOs-specific analyses, including adverse maternal outcomes (gestational diabetes, gestational hypertension, and premature rupture of membranes), failed newborn hearing tests, and neonatal jaundice. We not only investigated the exposure effects of daily mean temperature and diurnal air temperature variation but also daily maximum and minimum temperatures, to check the consistency of different heat metrics. Considering the possibly lagged effects of ambient temperature on APOs as reported by previous studies (6, 12), we generated heat exposure variables lagged for 12 weeks according to our preliminary cross-correlation analysis. All statistical analyses were performed by using Stata V17.0 (StataCorp LP, College Station, TX). The 0.05 level of statistical significance for two tails was adopted for all tests.

## 3. Results

**Figure 1:**
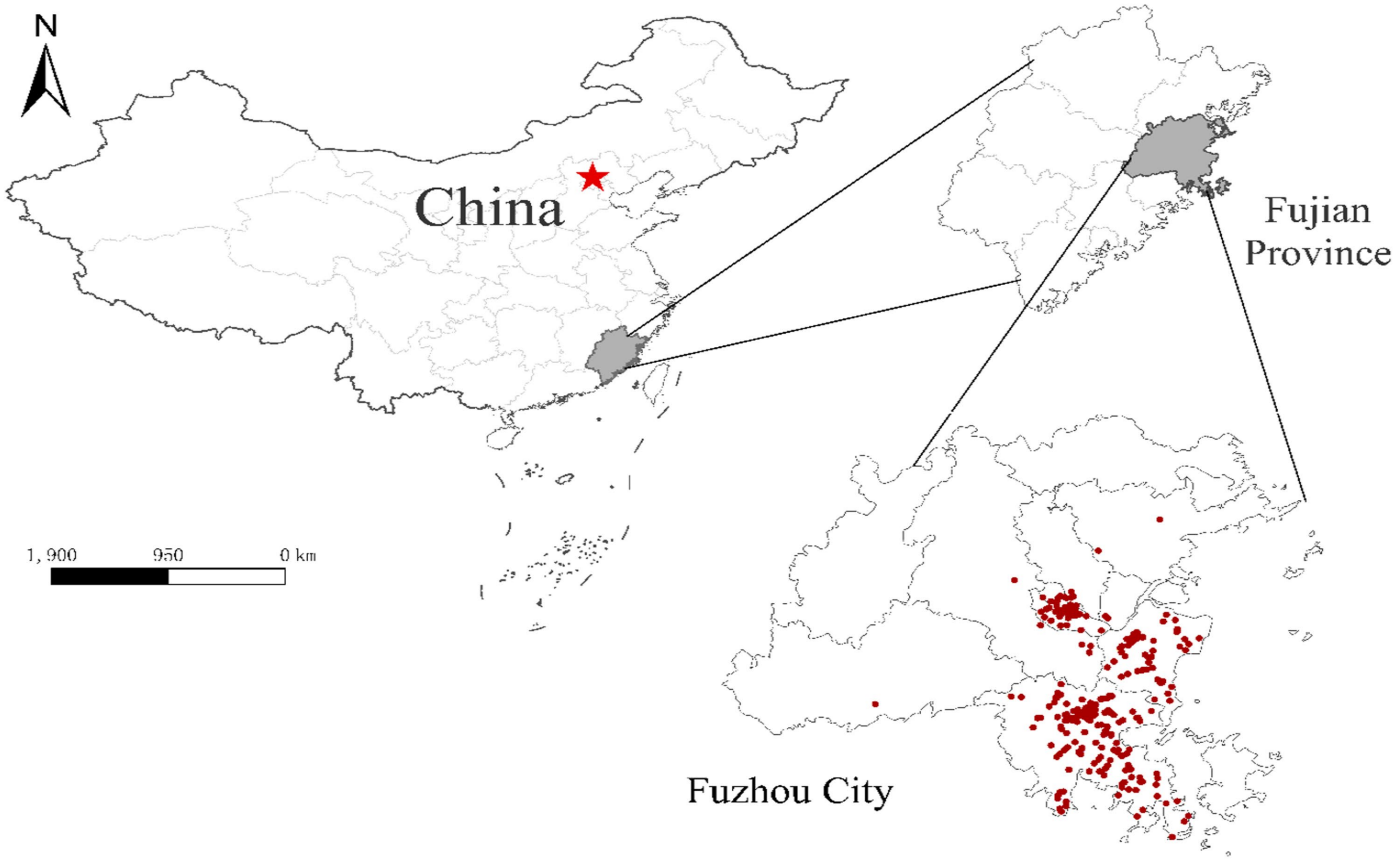
Location of Fuzhou City and the spatial distribution of participants. Red dots represent the residential addresses of participants. Red star indicates the capital city (Beijing) of China.

A total of 418 participants were recruited during the study period. The demographic characteristics of participants are summarized in Table 1 and Supplementary Table S1. Figure 1 shows the spatial distribution of the participants. Overall, there were 107 APOs, accounting for 25.6% of all participants. They include 23 participants suffering from pregnancy complications, 14 participants with high WBC, 37 infants who failed in hearing screening tests, 44 infants with neonatal jaundice, one each birth defect and low birth weight. Sixteen participants (14.9%) had more than one APOs. The vast majority (88.4%) of participants were less than 35 years old and the average maternal weight was 77.0 ± 8.4 kg. More than 80% of participants were recruited from the two suburban counties (Fuqing and Changle) of Fuzhou. About 40% of participants reported they were employed when being interviewed. Regarding household income, most participants fell into the range of 5,000–10,000 Yuan per month. About two-thirds of participants had two or more children. Twenty-one percent of participants got maternal infections, including rubella (75), syphilis (8), and other unspecified infections (6). In this study, 64.8% of infants were born in warm seasons. Vaginal birth accounted for 64.1%. The sex ratio (male/female) of infants was 0.92. The newborns’ average weight was 3253.6 ± 356.5 g. Results of bivariate analysis (Table 1) showed that pregnant women with a higher education level were at a lower risk of APOs, with the OR (95%CI) being 0.263 (0.088–0.784) for senior high school and 0.231 (0.077–0.692) for ≥undergraduate, compared to ≤primary school. Moreover, participants from the Fuzhou metro area were at lower risk of APOs than those from the suburban countries (OR = 0.228, 95%CI 0.102–0.513). Significant differences between APOs and Non-APOs were not observed by other covariates.

**Table 1:** Characteristics of the study participants.

Variables	Non-adverse pregnancy outcome	Adverse pregnancy outcome	OR (95%CI)	*p*-value
Total	311 (74.4)	107 (25.6)		
Maternal age (years)				
<25	58 (18.7)	24 (22.4)	-	-
25–29	132 (42.4)	35 (32.7)	0.641 (0.350–1.173)	0.149
30–34	85 (27.3)	33 (30.8)	0.938 (0.503–1.749)	0.841
≥35	36 (11.6)	15 (14.0)	1.007 (0.467–2.169)	0.986
Maternal weight (kg)	67.0 ± 8.3	66.9 ± 8.9	0.145	0.885
Baby weight (g)	3,257 ± 352	3,243 ± 370	0.352	0.725
Infant gender				
Female	152 (48.9)	60 (56.1)	-	-
Male	159 (51.1)	47 (43.9)	0.749 (0.481–1.165)	0.199
Gravidity				
1	93 (29.9)	35 (32.7)	-	-
2	110 (35.4)	36 (33.6)	0.870 (0.506–1.494)	0.613
3	69 (22.2)	24 (22.4)	0.924 (0.504–1.694)	0.799
≥4	39 (12.5)	12 (11.2)	0.818 (0.384–1.739)	0.601
Parity				
1	119 (38.3)	46 (43.0)	-	-
2	156 (50.2)	47 (43.9)	0.779 (0.486–1.249)	0.300
≥3	36 (11.6)	14 (13.1)	1.006 (0.497–2.036)	0.987
Occupation				
Not employed	182 (58.5)	67 (62.6)	-	-
Employed	129 (41.5)	40 (37.4)	0.842 (0.536–1.324)	0.457
Maternal education level				
Primary school or less	7 (2.3)	8 (7.5)	-	-
Junior high school	95 (30.6)	40 (37.4)	0.368 (0.125–1.084)	0.070
Senior high school	103 (33.1)	31 (29.0)	**0.263 (0.088–0.784)**	**0.017**
Undergraduate or more	106 (34.1)	28 (26.2)	**0.231 (0.077–0.692)**	**0.009**
Household income (Yuan per month)				
<3,000	16 (5.1)	2 (1.9)	-	-
3,000–5,000	101 (32.5)	40 (37.4)	3.168 (0.696–14.412)	0.136
5,000–10,000	143 (46.0)	37 (34.6)	2.070 (0.456–9.405)	0.346
≥10,000	42 (13.5)	18 (16.8)	3.429 (0.713–16.481)	0.124
Delivery method				
Vaginal birth	196 (63.0)	72 (67.3)	-	-
Cesarean	115 (37.0)	35 (32.7)	0.829 (0.520–1.319)	0.428
Type of feeding				
Formula feeding	155 (49.8)	57 (53.3)	-	-
Breastfeeding	156 (50.2)	50 (46.7)	0.872 (0.561–1.353)	0.540
Location				
Suburb	238 (76.5)	100 (93.5)	-	-
Metro	73 (23.5)	7 (6.5)	**0.228 (0.102–0.513)**	**0.000**
Infections				
No	248 (79.7)	82 (76.6)	-	-
Yes	63 (20.3)	25 (23.4)	1.200 (0.709–2.031)	0.497
Birth season				
Cold (Nov. to April)	104 (33.4)	43 (40.2)	-	-
Warm (May to Oct.)	207 (66.6)	64 (59.8)	0.748 (0.476–1.176)	0.208

Bold cells mean statistical significance. “-“refers to the reference group.

Characteristics of temperature exposure and PM_2.5_ during pregnancy by trimester were summarized in Table 2. The average temperature in the 2nd trimester was significantly lower than that in trimester one (Tukey-t = 9.88, 95%CI −4.94 to −3.05) and three (Tukey-t = −2.14, 95%CI −3.09 to −1.19), and similarly for maximum and minimum temperatures (data not shown). Temperature variations in the 3rd trimester were significantly higher than that in early (Tukey-t = 9.44, 95%CI 0.49 to 0.81) and middle (Tukey-t = 7.83, 95%CI 0.38 to 0.70) pregnancy. While for PM_2.5_, the highest average exposure was in the 2nd trimester, compared to trimester one (Tukey-t = 11.84, 95%CI 3.46 to 5.16) and three (Tukey-t = 1.95, 95%CI 1.10 to 2.80).

**Table 2:** Summary of daily temperature exposure and PM_2.5_ during pregnancy by gestational period.

	Mean ± SD	Maximum	Minimum	Percentile		
				25	50	75
Average temperature (°C)						
1st trimester	20.4 ± 6.3	28.7	9.5	14.6	21.9	26.4
2nd trimester	13.4 ± 5.1	26.5	9.0	11.6	15.5	20.8
3rd trimester	18.2 ± 6.0	26.8	9.1	12.3	17.5	24.5
Temperature variation (°C)						
1st trimester	5.4 ± 0.8	7.5	2.2	4.7	5.3	5.9
2nd trimester	5.5 ± 1.0	7.6	0.0	4.8	5.4	6.1
3rd trimester	6.0 ± 1.2	7.9	0.0	5.3	6.2	6.7
PM_2.5_ (μg/m^3^)						
1st trimester	27.0 ± 3.9	41.0	21.1	23.7	26.0	29.5
2nd trimester	31.8 ± 4.9	46.8	23.2	27.0	31.9	36.1
3rd trimester	29.4 ± 6.2	46.0	18.6	22.9	30.4	35.0

Table 3 shows the effects of prenatal temperature exposure on APOs at different pregnancy stages after controlling for potential confounders. We found overall a 1°C increase in mean temperature was associated with a 10.0% increase in APOs (aOR = 1.100, 95%CI 1.006–1.203) during the period of early pregnancy. However, APOs were negatively associated with temperature exposure in the middle pregnancy period, and a 1°C increase in mean temperature was associated 8.8% decrease in APOs (aOR = 0.912, 95%CI 0.846–0.982). Results of APOs-specific analysis show that infant jaundice was negatively associated with temperature exposure in the middle and late pregnancy periods. A 1°C increase in mean temperature was associated with 13.1% (aOR = 0.869, 95%CI 0.784–0.963) and 11.4% (aOR = 0.886, 95%CI 0.789–0.995) decrease of infant jaundice, respectively. With respect to the impacts of temperature on WBC count and newborns’ hearing, significant effects were observed only in the 3rd trimester. A 1°C increase in mean temperature may lead to a 1.8% (aOR = 0.981, 95%CI 0.857–1.122) decrease in failed newborn hearing screening and 29.6% (aOR = 1.296, 95%CI 1.019–1.649) increase in high WBC. A similar exposure-APOs pattern was observed in maximum and minimum temperatures (Supplementary Tables S2, S3).

**Table 3:** Effect estimates (odds ratio, 95% CI) of daily mean temperature on adverse pregnancy outcomes in Fuzhou.

Variables	Un-adjusted	Adjusted
1st trimester		
APOs	**1.050 (1.012–1.089)**	**1.100 (1.006–1.203)**
Pregnancy complications	**1.110 (1.021–1.208)**	1.108 (0.911–1.347)
Newborn hearing screening	0.994 (0.943–1.049)	0.984 (0.867–1.116)
Neonatal jaundice	**1.113 (1.047–1.184)**	1.123 (0.978–1.301)
High WBC	1.003 (0.922–1.092)	1.216 (0.987–1.499)
2nd trimester		
APOs	0.995 (0.953–1.039)	**0.912 (0.846–0.982)**
Pregnancy complications	1.032 (0.952–1.120)	0.918 (0.797–1.057)
Newborn hearing screening	0.948 (0.884–1.017)	0.938 (0.840–1.047)
Neonatal jaundice	1.019 (0.959–1.083)	**0.869 (0.784–0.963)**
High WBC	1.009 (0.910–1.120)	0.973 (0.821–1.153)
3rd trimester		
APOs	**0.948 (0.913–0.984)**	0.965 (0.884–1.052)
Pregnancy complications	**0.907 (0.838–0.982)**	0.959 (0.820–1.120)
Newborn hearing screening	1.013 (0.958–1.072)	0.981 (0.857–1.122)
Neonatal jaundice	**0.892 (0.840–0.948)**	**0.886 (0.789–0.995)**
High WBC	0.981 (0.898–1.073)	**1.296 (1.019–1.649)**

Bold cells mean statistical significance.

Table 4 shows the effects of diurnal temperature variation on APOs at the three trimesters after controlling for confounding factors. Overall, diurnal temperature variation had a significant impact on APOs in the third trimester and a 1°C increase in temperature variation was associated with a 35.1% (aOR = 1.351, 95%CI 1.023–1.784) increase in APOs. Results of APOs-specific analysis show that diurnal temperature variation had a significant impact on maternal WBC in the early and late pregnancy stages. A 1°C increase in temperature variation was associated with more than two times (aOR = 2.469, 95%CI 1.001–6.089) increase of high WBC in the first trimester and about five times (aOR = 4.724, 95%CI 1.548–14.409) increase in the third trimester.

**Table 4:** Effect estimates (odds ratio, 95% CI) of diurnal temperature variation on adverse pregnancy outcomes in Fuzhou.

Variables	Un-adjusted	Adjusted
1st trimester		
APOs	1.040 (0.799–1.355)	1.236 (0.834–1.831)
Pregnancy complications	1.353 (0.825–2.220)	1.044 (0.464–2.350)
Newborn hearing screening	0.743 (0.487–1.132)	0.731 (0.413–1.295)
Neonatal jaundice	1.198 (0.826–1.738)	1.202 (0.648–2.232)
High WBC	1.263 (0.674–2.368)	**2.469 (1.001–6.089)**
2nd trimester		
APOs	**0.715 (0.569–0.900)**	0.820 (0.618–1.088)
Pregnancy complications	0.714 (0.500–1.023)	0.690 (0.433–1.100)
Newborn hearing screening	0.960 (0.684–1.347)	0.989 (0.688–1.422)
Neonatal jaundice	**0.594 (0.439–0.806)**	0.633 (0.440–0.910)
High WBC	0.818 (0.505–1.327)	0.949 (0.516–1.744)
3rd trimester		
APOs	0.913 (0.773–1.121)	**1.351 (1.023–1.784)**
Pregnancy complications	0.834 (0.609–1.142)	0.994 (0.629–1.567)
Newborn hearing screening	1.089 (0.799–1.484)	1.202 (0.780–1.808)
Neonatal jaundice	**0.790 (0.624–1.000)**	1.035 (0.741–1.446)
High WBC	1.440 (0.809–2.563)	**4.724 (1.548–14.409)**

Bold cells mean statistical significance.

**Figure 2:**
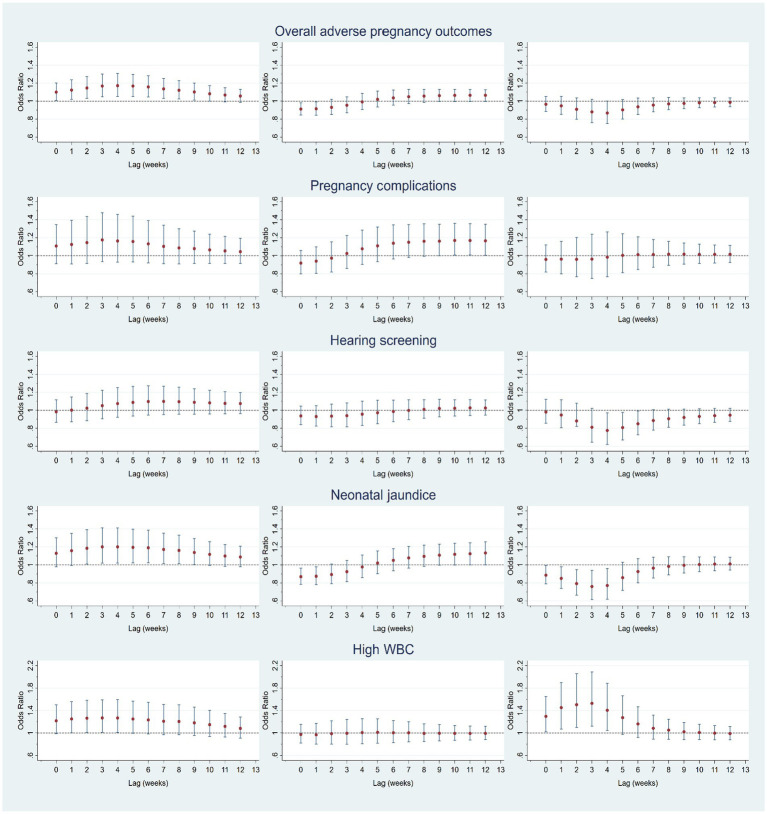
Lagged effects (0–12 weeks) of mean temperature on overall adverse pregnancy outcomes (APOs) and APOs-specific diagnoses (maternal complications, failed hearing screening, neonatal jaundice, and high WBC) at trimester 1 (left column), trimester 2 (middle column), and trimester 3 (right column).

Figure 2 shows the lagged effects of mean temperature on overall APOs and diagnosis-specific conditions by trimesters after controlling for confounding factors. For overall APOs in trimester 1, we found the lagged effects of ambient temperature last 10 weeks, with the greatest effects (aOR = 1.172, 95%CI 1.051–1.306) observed at lag week 4, while significantly lagged effects were not observed in trimesters 2 and 3. Results of diagnosis-specific analysis show that mean temperature had no lagged effects on complications of pregnancy (gestational diabetes, gestational hypertension, and premature rupture of membranes) during the entire pregnancy period. For newborn hearing screening, lagged effects were not observed in the early and middle stages of pregnancy. However, the mean temperature was negatively associated with the risk of a failed hearing screening test at lags 4–6 weeks, with the greatest effect (aOR = 0.776, 95%CI 0.619–0.973) observed at lag week 4. The risk of neonatal jaundice increased at lag weeks 2–9 in the first trimester, with the greatest lagged effect (aOR = 1.201, 95%CI 1.020–1.413) observed at lag week 3. However, the risk of neonatal jaundice significantly reduced at lag weeks 0–1 in trimester 2 and lag weeks 0–4 in trimester 3. The risk of high WBC significantly increased at lag weeks 1–4 in the first trimester with the greatest effect observed at lag week 3 (aOR = 1.268, 95%CI 1.010–1.590). By contrast, the risk of high WBC last 4 weeks in the third trimester with the greatest effect observed at lag week 4 (aOR = 1.404, 95%CI 1.046–1.883).

## 4. Discussion

With the predicted likely increasing frequency and intensity of extreme temperatures, climate change is presenting a growing challenge for maternal, fetal, and neonatal health (4, 9, 10). In this study, we examined the impact of ambient temperature on some under-researched APOs including maternal complications, high WBC, newborn hearing loss, and neonatal jaundice. The findings of this study may not only expand the spectrum of temperature-attributable APOs but also provide insights for mitigating the health-related harms of a warming climate on pregnant women, fetuses, and newborns.

In this study, we found APOs were positively associated with mean temperatures in the 1st trimester with lagged effects lasting up to 10 weeks. However, a negative temperature-APOs association was observed in the 2nd trimester with a 3-week lagged effect. Generally, findings of previous studies suggest that the last few weeks of pregnancy were more sensitive to APOs (6, 27). Our results show that diurnal temperature variation significantly increased the risk of APOs in the 3rd trimester. The discrepancy between different heat metrics needs further investigation. The temperature-APOs and susceptible windows vary depending on the outcomes being examined. Therefore, we conducted APOs-specific analyses.

Neonatal jaundice is characterized with yellowish discoloration of the sclera and skin in a newborn baby due to the accumulation of unconjugated bilirubin. Neonatal jaundice occurs in up to 85% of all live birth (28). Most neonatal jaundice is self-limiting and does not require special treatment. Recent studies even suggest that unconjugated bilirubin may play a protective role against oxidative stress (29). However, severe neonatal jaundice may not only lead to infants’ long-term neurological impairments but also affect mothers’ psychological health (30, 31). Multiple factors have been identified associated with neonatal jaundice such as ethnicity, geography, gender of newborns, birth weight, gestational age, labor and delivery, blood group incompatibility, eating habits, and genetics (28). Since Milby et al. observed a seasonal variation in the incidence of neonatal jaundice among 3,096 babies delivered in an agricultural community hospital in California during 1963–1966 (32), dozens of studies investigated the influence of birth season or ambient temperature on the risk of neonatal jaundice (33). However, there is no consensus and the potential mechanisms of seasonal fluctuations in the development of neonatal jaundice remain unclear.

In this study, we found neonatal jaundice was negatively associated with ambient temperature at trimesters 2 and 3. This is consistent with evidence from Japan (33), Finland (34), and Iran (35). The relatively short duration of daylight during the cold season may increase the risk of hyperbilirubinemia, as the sun emits blue–green light in the spectrum needed to most effectively convert bilirubin to its water-soluble isomers for excretion (36). In addition, parents usually increase indoor temperatures to prevent newborns from hypothermia, which can cause dehydration and increase serum bilirubin levels (35). However, a recent comprehensive literature review found more studies tend to support a positive relationship between birth season and neonatal jaundice (33). The possible explanations include: (1) Neonates tend to drink more breast milk during the hot season to compensate for dehydration. This may contribute to the development of neonatal jaundice, as human milk inhibits the conjugation of bilirubin (28); (2) During hot summer, parents are usually reluctant to be outdoors which may reduce the potential for phototherapy; and (3) Neonatal jaundice is relatively easier to be found as newborns wear fewer clothes in the hot season. Divergent findings in the previous studies may be due to the differences in case inclusion criteria, study design, and various cut-offs for hyperbilirubinemia (28, 37). We noticed that previous studies did not explore the possible lagged effects of heat exposure on neonatal jaundice. Moreover, we found a positive relationship between mean temperature and neonatal jaundice at lag weeks 2–9 in trimester 1, while the lagged effects were negative in trimester 2 (lag week 1) and 3 (lag weeks 1–4). This may be another important factor resulting in the inconsistencies in previous studies and deserves further research on the molecular mechanisms of pathogenesis.

Infections during pregnancy are associated with a wide range of morbidity and mortality in pregnant women and developing fetus, such as maternal death, congenital anomalies, pregnancy loss, preterm birth, low birth weight, and even lifelong consequences to the neonates (38). White blood cells are commonly measured in pregnancy as an indicator to facilitate the diagnosis of infection or inflammation in clinical practice. Evidence has shown that ambient temperatures were associated with infections-related morbidity and mortality in the general population (39). Compared with non-pregnant women, pregnant women are more likely to be severely affected by infections (40). However, few studies investigated the impact of ambient temperature on infections during pregnancy. In this study, we found that mean temperatures were positively associated with high WBC in the third trimester with lagged effects lasting for up to 4 weeks. Moreover, temperature variations had a significant impact on high WBC at trimesters one and three. The increase in temperature may accelerate the incubation period of microorganisms and the population dynamics of vector species and pathogenic organisms. Warmer temperatures can lead to increased proliferation of pathogens in food and water sources (41). Therefore, temperature variation may be an independent risk factor leading to infections during pregnancy. This deserves more attention, especially in the context of global warming. There is a need to strengthen the awareness of pregnant women and relevant stakeholders about the prevention of infections during warm days for protecting maternal and infant health.

Although the long-term health effects of extreme heat exposure on maternal complications are largely unknown, mounting evidence shows that elevated ambient temperatures were associated with adverse maternal outcomes such as gestational diabetes mellitus, hypertensive disorders, premature rupture of membrane, placental abruption, maternal stress, and cardiovascular risk at labor (6). Currently, the physiological mechanisms that underpin these associations are poorly understood, which may include reduced placental blood flow, oxidative stress and release of inflammatory markers, heat shock proteins, dehydration, uterine contractility, and hypercoagulability (42). However, in this study daily mean temperature and diurnal temperature variation had no significant impacts on maternal outcomes, probably due to the small sample size which could not generate sufficient statistical power. We also explored the impact of ambient temperature on newborn hearing loss, as there is evidence that the increase in body core temperature could result in transient deafness among infants (43). We did not observe a significant temperature-hearing loss association at lag 0, but a negative association was found at lag weeks 4–6. Herbig examined the effects of ambient temperature and relative humidity on the initial refer rate from January to March 2003 in newborn hearing screening using transient evoked otoacoustic emissions testing and did not find statistically significant results (44).

This study has several limitations that should be acknowledged. Firstly, the relatively small sample size in this study dictates a cautious interpretation of the results, although it is a common issue in temperature-APOs research (6). Therefore, future studies with a big sample size are warranted for the causal inference between temperature and APOs. Secondly, this study was only conducted in one city (Fuzhou), which may limit the generalizability of the findings to other regions or populations with different demographical characteristics. Thirdly, the study relied on self-reported data from participants which may introduce recall bias or measurement errors, although relevant quality assurance and quality control procedures have been adopted during the survey. Fourthly, recent evidence shows that temperature extremes can independently and act synergistically with PM_2.5_ on APOs (45). In this study, we did not explore the interactive effects between ambient temperature and PM_2.5_ on APOs. Under a warming climate, adverse pregnancy outcomes attributed to climate change-driven increases in heat exposure and air pollution may be larger than considering these risk factors individually (46).

## 5. Conclusion

Pregnant women, fetuses, and newborns are vulnerable to extreme temperatures, which may be exaggerated by the ongoing climate crisis. A diverse range of heat-related adverse pregnancy outcomes has been reported, but less attention has been paid to heat-attributed neonatal jaundice, newborn hearing loss, and infections during pregnancy. Women’s healthcare providers can educate their patients about the adverse health effects of high temperatures. In addition to the identified epidemiologic link and susceptible exposure windows, there is a need to understand the underlying biological mechanisms for better recommendations for climate change adaptation policies.

## Data availability statement

The raw data supporting the conclusions of this article will be made available by the authors, without undue reservation.

## Ethics statement

The studies involving human participants were reviewed and approved by Ethics Committees of Fuzhou Maternity & Child Healthcare Hospital (20151110FQ) Ethics Committees of Fuqing Women & Children Hospital (20151110FZ) Ethics Committees of Changle Hospital (20160307). The patients/participants provided their written informed consent to participate in this study.

## Author contributions

JX, YX, RX, and JFL conceived and designed the study. YY, AN, TY, JFL, ZZ, and JX analyzed the data. JX and JFL drafted the manuscript. JX, JWL, YC, QL, CW, BL, RX, and YX revised and edited the manuscript. All authors read and approved the final manuscript.

## Funding

The study was funded by the Natural Science Foundation of Fujian Province (2021J01722) and Fuzhou Health Technology Project (2019-S-wp7).

## Conflict of interest

The authors declare that the research was conducted in the absence of any commercial or financial relationships that could be construed as a potential conflict of interest.

## Publisher’s note

All claims expressed in this article are solely those of the authors and do not necessarily represent those of their affiliated organizations, or those of the publisher, the editors and the reviewers. Any product that may be evaluated in this article, or claim that may be made by its manufacturer, is not guaranteed or endorsed by the publisher.

## Footnotes

^1^ https://zenodo.org/record/6398971#.Yrz8NBVBzGL

^2^ https://lbsyun.baidu.com/apiconsole/user/choose
