# Supplementary material for: Impact of ambient temperature on adverse pregnancy outcomes: a birth cohort study in Fuzhou, China
Source: Front Public Health. 2023 Jul 6;11:1183129. doi: 10.3389/fpubh.2023.1183129 (PMC10359494; doi:10.3389/fpubh.2023.1183129)
Supplement: Supplementary file 1 [file Table_1.docx]

| **Table 1: Characteristics of study participants by different adverse pregnancy outcomes** | | | | | |
| --- | --- | --- | --- | --- | --- |
| Variable | Pregnancy complications | Newborn hearing screening | Neonatal jaundice | High WBC count | Total |
| Total | 23 | 37 | 44 | 14 | 107 |
| Maternal age (yrs) |  |  |  |  |  |
| <25 | 7(30.4) | 9(24.3) | 9(20.5) | 2(14.3) | 58(18.7) |
| 25-29 | 6(26.1) | 12(32.4) | 18(40.9) | 5(35.7) | 132(42.4) |
| 30-34 | 7(30.4) | 12(32.4) | 11(25.0) | 5(35.7) | 85(27.3) |
| ≥35 | 3(13.0) | 4(10.8) | 6(13.6) | 2(14.3) | 36(11.6) |
| Maternal weight (kg) | 67.4±7.8 | 67.6±9.9 | 67.1±7.3 | 65.4±12.4 | 77±8.4 |
| Baby weight (g) | 3315.4±410.2 | 67.7±4.6 | 3217.1±376.8 | 3172.1±338.8 | 3253.6±356.5 |
| Infant gender |  |  |  |  |  |
| Female | 12(52.2) | 25(67.6) | 19(43.2) | 8(57.1) | 212(50.7) |
| Male | 11(47.8) | 12(32.4) | 25(56.8) | 6(42.9) | 206(49.3) |
| Gravidity |  |  |  |  |  |
| 1 | 10(43.65) | 10(27.0) | 20(45.5) | 5(35.7) | 128(30.6) |
| 2 | 7(30.4) | 15(40.5) | 10(22.7) | 4(28.6) | 146(34.9) |
| 3 | 5(21.7) | 8(21.6) | 7(15.9) | 5(35.7) | 93(22.3) |
| ≥4 | 1(4.4) | 4(10.8) | 7(15.9) | 0(0.0) | 51(12.2) |
| Parity |  |  |  |  |  |
| 1 | 15(65.2) | 13(35.1) | 22(50.0) | 6(42.9) | 165(39.5) |
| 2 | 5(21.7) | 18(48.7) | 17(38.6) | 8(57.1) | 203(48.6) |
| ≥3 | 3(13.1) | 6(16.2) | 5(11.4) | 0(0.0) | 50(12.0) |
| Occupation |  |  |  |  |  |
| Not employed | 12(52.2) | 25(67.6) | 28(63.6) | 6(42.9) | 249(59.6) |
| Employed | 11(47.8) | 12(32.4) | 16(36.4) | 8(57.1) | 169(40.4) |
| Education level |  |  |  |  |  |
| Primary school or less | 1(4.4) | 2(5.4) | 4(9.1) | 1(7.1) | 15(3.6) |
| Junior high school | 7(30.4) | 18(48.7) | 15(34.1) | 5(35.7) | 135(32.3) |
| Senior high school | 6(26.1) | 12(32.4) | 13(29.6) | 3(21.4) | 134(32.1) |
| Undergraduate or more | 9(39.1) | 5(13.5) | 12(27.3) | 5(35.7) | 134(32.1) |
| Household income (Yuan per month) | |  |  |  |  |
| <3,000 | 0(0.0) | 0(0.0) | 2(4.6) | 0(0.0) | 18(4.3) |
| 3,000-5,000 | 10(43.5) | 18(48.7) | 13(29.6) | 8(57.1) | 141(33.7) |
| 5,000-10,000 | 9(39.1) | 11(29.7) | 15(34.1) | 4(28.6) | 180(43.1) |
| ≥10,000 | 4(17.4) | 6(16.2) | 6(13.6) | 2(14.3) | 60(14.4) |
| Delivery method |  |  |  |  |  |
| Vaginal birth | 14(60.9) | 27(73.0) | 29(65.9) | 10(71.4) | 268(64.1) |
| Caesarean | 9(39.1) | 10(27.0) | 15(34.1) | 4(28.6) | 150(35.9) |
| Type of feeding |  |  |  |  |  |
| Formula feeding | 11(47.8) | 25(67.6) | 16(36.4) | 11(78.6) | 212(50.7) |
| Breastfeeding | 12(52.2) | 12(32.4) | 28(63.6) | 3(21.4) | 206(49.3) |
| Location |  |  |  |  |  |
| Suburb | 21(91.3) | 32(86.5) | 44(100.0) | 14(100.0) | 338(80.9) |
| Metro | 2(8.7) | 5(13.5) | 0(0.0) | 0(0.0) | 80(19.1) |
| Maternal infections |  |  |  |  |  |
| No | 16(69.6) | 27(73.0) | 34(77.3) | 12(85.7) | 330(79.0) |
| Yes | 7(30.4) | 10(27.0) | 10(22.7) | 2(14.3) | 88(21.0) |
| Birth season |  |  |  |  |  |
| Cold (Nov. to April) | 12(52.2) | 11(29.7) | 21(47.7) | 5(35.7) | 147(35.2) |
| Warm (May to Oct.) | 11(47.8) | 26(70.3) | 23(52.3) | 9(64.3) | 271(64.8) |

| **Table 2: Effect estimates (odds ratio, 95% CI) of daily maximum temperature on adverse pregnancy outcomes in Fuzhou** | | | |
| --- | --- | --- | --- |
| Variables | Un-adjusted |  | Adjusted |
| 1^st^ trimester |  |  |  |
| APOs | 1.049(1.011-1.088) |  | 1.085(0.993-1.184) |
| Pregnancy complications | 1.107(1.201-1.207) |  | 1.091(0.905-1.316) |
| Newborn hearing screening | 0.990(0.937-1.045) |  | 0.965(0.855-1.089) |
| Neonatal jaundice | 1.106(1.042-1.174) |  | 1.100(0.958-1.263) |
| High WBC count | 1.016(0.931-1.108) |  | 1.280(1.028-1.593) |
| 2^nd^ trimester |  |  |  |
| APOs | 0.970(0.925-1.018) |  | 0.902(0.842-0.967) |
| Pregnancy complications | 1.003(0.916-1.098) |  | 0.901(0.793-1.024) |
| Newborn hearing screening | 0.937(0.868-1.011) |  | 0.935(0.845-1.033) |
| Neonatal jaundice | 0.982(0.917-1.052) |  | 0.862(0.784-0.949) |
| High WBC count | 1.005(0.896-1.127) |  | 0.983(0.844-1.145) |
| 3^rd^ trimester |  |  |  |
| APOs | 0.955(0.924-0.988) |  | 1.003(0.937-1.074) |
| Pregnancy complications | 0.918(0.857-0.984) |  | 0.965(0.856-1.089) |
| Newborn hearing screening | 1.011(0.962-1.063) |  | 0.995(0.898-1.102) |
| Neonatal jaundice | 0.907(0.860-0.956) |  | 0.927(0.848-1.012) |
| High WBC count | 0.995(0.920-1.076) |  | 1.283(1.040-1.583) |

| **Table 3: Effect estimates (odds ratio, 95% CI) of daily minimum temperature on adverse pregnancy outcomes in Fuzhou** | | | |
| --- | --- | --- | --- |
| Variables | Un-adjusted |  | Adjusted |
| 1^st^ trimester |  |  |  |
| APOs | 1.059(1.016-1.103) |  | 1.099(0.993-1.216) |
| Pregnancy complications | 1.129(1.028-1.241) |  | 1.123(0.903-1.396) |
| Newborn hearing screening | 0.994(0.936-1.055) |  | 0.972(0.847-1.117) |
| Neonatal jaundice | 1.131(1.055-1.212) |  | 1.120(0.957-1.312) |
| High WBC count | 1.014(0.921-1.116) |  | 1.272(1.009-1.603) |
| 2^nd^ trimester |  |  |  |
| APOs | 0.985(0.938-1.034) |  | 0.888(0.820-0.962) |
| Pregnancy complications | 1.022(0.932-1.121) |  | 0.897(0.774-1.039) |
| Newborn hearing screening | 0.936(0.865-1.012) |  | 0.921(0.823-1.031) |
| Neonatal jaundice | 1.009(0.942-1.081) |  | 0.852(0.764-0.950) |
| High WBC count | 1.016(0.904-1.142) |  | 0.982(0.826-1.168) |
| 3^rd^ trimester |  |  |  |
| APOs | 0.948(0.914-0.984) |  | 0.976(0.900-1.056) |
| Pregnancy complications | 0.908(0.840-0.981) |  | 0.953(0.825-1.099) |
| Newborn hearing screening | 1.011(0.957-1.068) |  | 0.975(0.865-1.098) |
| Neonatal jaundice | 0.896(0.844-0.950) |  | 0.900(0.806-0.998) |
| High WBC count | 0.983(0.902-1.073) |  | 1.213(1.003-1.167) |
